# Immunization with mRNA-LNPs Encoding *Ornithodoros* Argasid Tick Antigens Induces Humoral Immune Responses and Tick Resistance

**DOI:** 10.3390/pathogens14090914

**Published:** 2025-09-11

**Authors:** Ana Oleaga, Ana Laura Cano-Argüelles, María González-Sánchez, Rocío Vizcaíno-Marín, Ricardo Pérez-Sánchez

**Affiliations:** Parasitology Laboratory, Institute of Natural Resources and Agrobiology (IRNASA-CSIC), Cordel de Merinas, 40-52, 37008 Salamanca, Spain; ana.oleaga@irnasa.csic.es (A.O.); ana.cano@irnasa.csic.es (A.L.C.-A.); m.g@csic.es (M.G.-S.); rocio.vizcaino@irnasa.csic.es (R.V.-M.)

**Keywords:** *Ornithodoros erraticus*, *Ornithodoros moubata*, mRNA vaccines, lipid nanoparticles, salivary antigens, midgut antigens

## Abstract

Argasid ticks *Ornithodoros erraticus* and *Ornithodoros moubata* are major vectors of zoonotic pathogens, including the African swine fever virus and relapsing fever *Borrelia* spp., and their control is essential to reduce disease transmission. In this study, we evaluated the immunogenicity and protective efficacy of four *Ornithodoros* tick antigens formulated as mRNA–lipid nanoparticles (mRNA-LNPs): OeSOD, OeTSP1, OmPLA2, and Om86. Rabbits were immunised with three doses of each mRNA-LNP construct, and immune responses and tick biological parameters were assessed following infestation with both tick species. All mRNA-LNP constructs induced antigen-specific IgG responses that recognised native proteins in tick saliva and midgut extracts. Vaccination resulted in significant reductions in female oviposition and fertility, which correlated with antibody levels, and yielded protective efficacies of 21.9–41.6% against *O. moubata* and 23.1–41.6% against *O. erraticus*. Notably, the mRNA-LNPs of OeSOD and OeTSP1 outperformed their recombinant counterparts against *O. moubata*, and Om86 mRNA-LNP conferred markedly improved protection against both *O. moubata* and *O. erraticus*. These findings highlight the potential of mRNA-LNP vaccines to induce effective anti-argasid tick immunity and provide a promising platform for the development of sustainable strategies to control argasid ticks and associated pathogens.

## 1. Introduction

Ticks are obligate haematophagous ectoparasites classified into two principal families: Ixodidae (hard ticks) and Argasidae (soft ticks). Ixodid ticks are typically exophilic, dwelling in soil and vegetation and actively seeking hosts during favourable environmental conditions. Upon host attachment, they undergo prolonged feeding lasting several days, during which they ingest large volumes of blood. Once fully engorged, they detach and return to the environment, where they moult or, in the case of females, oviposit and subsequently die. In contrast, argasid ticks exhibit endophilic or nidicolous behaviour. In natural settings, they reside within the nests and burrows of their hosts, while in synanthropic environments, they colonise animal shelters and human dwellings, hiding in crevices within floors, walls, and ceilings. These protected environments provide them with shelter from adverse climatic conditions and consistent access to vertebrate hosts. Most argasids are fast feeders, completing blood meals within an hour. After feeding, they detach and return to sheltered sites to moult or reproduce. Adult argasid ticks are capable of multiple feeding and reproductive cycles and display remarkable resistance to starvation, surviving for several years without a blood meal [1].

Ticks constitute an increasing concern in both human and veterinary medicine due to the direct damage caused by feeding, but more significantly due to their role as efficient vectors of a wide array of pathogens. These pathogens are responsible for numerous diseases affecting wildlife, livestock, companion animals, and humans, leading to substantial economic losses globally [2].

Within the Argasidae, *Ornithodoros erraticus* and *Ornithodoros moubata* are recognised vectors of major zoonotic and epizootic pathogens. *O. erraticus* is a key vector of the African swine fever (ASF) virus and tick-borne relapsing fever (TBRF) spirochaetes (*Borrelia* spp.) in the Mediterranean Basin and the middle East, while *O. moubata* plays a similar role in sub-Saharan Africa [3,4,5,6,7,8]. Specifically, *O. erraticus* has been associated with *Borrelia hispanica* in the Iberian Peninsula and Northern Africa [7] and with *Borrelia microtti* in Iran, Afghanistan and Eastern Africa [8], whereas *O. moubata* has been linked to *Borrelia duttoni* in Central, Eastern and Southern Africa [8]. Moreover, *O. moubata* has recently been identified as a competent vector of *Coxiella burnetii*, the causative agent of Q fever [9].

The persistence of these *Ornithodoros* species in anthropized environments significantly contributes to the maintenance of ASF and TBRF in endemic regions and may facilitate their dissemination into adjacent territories. Consequently, any comprehensive strategy aimed at the prevention and control of these diseases should, at a minimum, incorporate measures to control *Ornithodoros* vector populations in human-influenced environments.

Current tick control strategies rely predominantly on the use of chemical acaricides. However, the widespread and often indiscriminate application of these compounds has led to the emergence of acaricide-resistant tick populations and the accumulation of chemical residues in animal-derived products and the environment. These drawbacks underscore the urgent need for alternative, sustainable methods of tick control [10,11]. In this context, anti-tick vaccines have emerged as a promising, environmentally sustainable, and effective strategy for controlling tick infestations and reducing the transmission of tick-borne diseases [12,13].

A major challenge in the development of effective anti-tick vaccines is the identification of suitable protective antigens. Currently, two main methodological approaches are employed. The first is a rational strategy based on detailed knowledge of tick biology, in which proteins essential for tick survival, feeding, or reproduction are selected as candidate antigens. The second approach is vaccinomics-based and involves hypothesis-driven selection of vaccine candidates from omics datasets, which are generated through the analysis of tick–host–pathogen interactions [14,15]. Notably, a novel strategy for identifying antigenic candidates for tick vaccines is the development of *Ixodes scapularis* rapid extracellular antigen monitoring (IscREAM), a technique that detects antibody responses to over 3000 tick antigens. This approach has identified several immunogenic antigens, enhancing the understanding of the host immune response and paving the way for future anti-tick vaccine development [16].

Recent advances in vaccinomics have accelerated the identification of candidate antigens in *O. erraticus* and *O. moubata*. These include proteins expressed in the salivary glands and midgut tissues of both species [17,18,19,20]. Several of these antigens have been shown to be immunogenic and to confer partial protection against *O. erraticus* and/or *O. moubata* in animal models when delivered as recombinant proteins [21,22,23,24,25]. Nonetheless, substantial improvements are still needed to enhance both the magnitude and duration of the immune responses induced by these vaccines. The protective effects of most anti-tick vaccines are believed to result from antibody-mediated neutralisation or functional inhibition of essential tick proteins. Therefore, more robust and long-lasting antibody responses are generally associated with higher levels of vaccine-induced protection.

While protein-based vaccines have formed the foundation of immunisation strategies over the past century, recent advances in vaccinology have highlighted the potential of vaccines based in mRNA formulated in lipid nanoparticles (mRNA-LNPs). These vaccines deliver nucleotide sequences encoding the target antigen into host cells, where they are translated in situ into proteins. These endogenously generated antigens are then processed and presented to the immune system, eliciting both humoral (B cell-mediated) and cellular (T cell-mediated) responses, which are critical for effective and durable immunity. Importantly, mRNA-LNP vaccines may overcome challenges associated with recombinant protein production, such as difficulties in achieving correct folding or post-translational modifications, particularly glycosylation. By enabling the host to produce antigens in their native form, mRNA-LNP vaccines can preserve conformational and glycosylation-dependent epitopes that are frequently lost during expression in prokaryotic systems. This structural fidelity is expected to enhance immunogenicity and, ultimately, protective efficacy [26,27,28,29,30].

Pioneering research on mRNA-based anti-tick vaccines has recently focused on the ixodid tick *Ixodes scapularis*, a well-established vector of *Borrelia burgdorferi*, the causative agent of Lyme disease. Matias et al. [31] compared mRNA, DNA, and recombinant protein platforms for delivery of Salp14, a tick salivary anticoagulant protein. Their findings showed that the mRNA-based vaccine induced the strongest antibody responses and most prominent erythema at the tick bite site in guinea pigs, which are hallmarks of acquired tick resistance. This study demonstrated the feasibility and superiority of mRNA-LNP vaccines in the context of tick-borne disease prevention. Subsequent work by the same group involved mRNA-LNP cocktail vaccines encoding up to 19 *I. scapularis* salivary proteins (referred to as 19ISP), which were selected based on their roles in promoting host inflammation or inhibiting *Borrelia* transmission. Vaccinated guinea pigs mounted strong antibody responses, and nymphal ticks fed on these animals exhibited impaired feeding and early detachment. Furthermore, the 19ISP vaccine blocked transmission of *B. burgdorferi* in this model [32,33]. More recently, administration of 19ISP to rabbits elicited strong humoral responses that, while not impairing adult tick feeding, significantly reduced female fecundity, indicating potential for disrupting the tick reproductive cycle [34]. Together, these studies validate the use of mRNA-LNP platforms in targeting ixodid tick salivary antigens and offer a foundation for the development of novel strategies for tick control.

Building on this promising evidence, the present study aimed to explore the potential of mRNA-LNP vaccines for targeting *Ornithodoros* ticks. We selected four antigens from *Ornithodoros* spp. that had previously demonstrated partial protective efficacy against *O. erraticus* and/or *O. moubata* when administered as recombinant proteins. These antigens were produced in an mRNA-LNP format and used to immunise rabbits. Our objective was to compare the immunogenicity and protective efficacy of the mRNA-LNP formulations with those of their homologous recombinant protein counterparts, in order to assess the potential advantages of the mRNA-LNP platform for the development of vaccines against argasid ticks.

## 2. Materials and Methods

### 2.1. Ticks and Tick Material

The *O. erraticus* and *O. moubata* ticks used in this study came from the laboratory colonies kept at IRNASA (CSIC), Spain. The *O. erraticus* colony was set from specimens captured in nature in the Salamanca province (Spain), whereas the *O. moubata* colony was established from specimens originating from Malawi, kindly provided by the Institute for Animal Health in Pirbright (Surrey, UK). These ticks are regularly fed on New Zealand white rabbits and maintained at 28 °C, 85% relative humidity and a 12 h light–dark cycle.

Saliva samples from *O. erraticus* and *O. moubata* females were obtained by stimulating salivation with pilocarpine, following the protocol described by Cano-Argüelles et al. [25]. Ticks were sequentially washed in running tap water, 3% hydrogen peroxide, distilled water (twice), 70% ethanol, and again twice in distilled water. After drying, ticks were immobilised ventral side up on a glass plate using double-sided adhesive tape, in groups of five individuals. Each tick received 1 µL of 1% pilocarpine hydrochloride (Sigma) in phosphate-buffered saline (PBS), administered with a 5 µL Hamilton syringe fitted with a 33-gauge, 25 mm needle. Salivation was induced within minutes, characterised by movement of the chelicerae and the secretion of small droplets of clear, viscous saliva. To collect the saliva, 1 µL of PBS was placed on the tick’s mouthparts to dilute each droplet, immediately aspirated, and transferred into 50 µL of ice-cold PBS. Saliva collection continued for 15–20 min post-stimulation. The pooled saliva samples were then centrifuged at 12,000× *g* for 20 min at 4 °C, and the resulting supernatants were recovered. Protein concentration was determined using BCA Protein Assay Reagent Kit (Thermo Fisher, Madrid, Spain), and the samples were stored at −20 °C until use.

Midguts were dissected from fasted *O. erraticus* and *O. moubata* females and used to prepare midgut protein extracts according to the protocol described by Oleaga et al. [17]. Batches of 25 midguts per species were suspended in ice-cold PBS supplemented with protease inhibitors (Roche Diagnostics, San Cugat del Vallés, Barcelona, Spain), homogenised on ice using an Ultra-Turrax T10 disperser (IKA-Werke Spain, Barcelona, Spain), and sonicated six times for 60 s each in ice-cold PBS. The homogenates were then centrifuged at 10,000× *g* for 20 min at 4 °C to remove cellular debris, and the resulting supernatants were collected. Protein concentrations were determined using the BCA Protein Assay Reagent Kit, and the extracts were stored at −20 °C until use.

### 2.2. Antigen Selection and Formulation of mRNA-Lipid Nanoparticles (mRNA-LNPs)

Four vaccine candidate antigens from *Ornithodoros* ticks—one salivary and one intestinal antigen from each species—were selected for formulation as mRNA-LNPs: OeSOD and OeTSP1 from *O. erraticus*, and OmPLA2 and Om86 from *O. moubata*. These antigens were chosen based on their previously demonstrated partial protective efficacy against *O. erraticus* and/or *O. moubata* in rabbits when administered as recombinant proteins, thereby leaving scope for further improvement (Table 1). Firefly (*Photinus pyralis*) luciferase was included as an unrelated control antigen.

mRNA-LNPs encoding tick antigens and control firefly luciferase were produced by GenScript (Rijswijk, The Netherlands). Briefly, template plasmids containing codon-optimised sequences for expression in rabbits, encoding either tick antigens or luciferase, were generated and subsequently linearized. In vitro transcription was performed to synthesise mRNAs bearing a 100-nucleotide poly(A) tail. To incorporate modified nucleosides, N1-methylpseudouridine-triphosphate was used in place of UTP. Co-transcriptional 5′ capping was achieved using the trinucleotide cap 1 analogue, CleanCap. The resulting mRNAs were purified using a silica membrane-based method and assessed for size and integrity by capillary electrophoresis.

The mRNAs were encapsulated in LNPs using the ionisable cationic lipid SM-102 through a self-assembly process (GenScript, Rijswijk, The Nederlands). The resulting LNPs exhibited diameters ranging from 77 to 87 nm, with a polydispersity index (PDI) of 0.07–0.09, as determined by dynamic light scattering (Zetasizer). Encapsulation efficiency, measured using the RiboGreen assay, ranged from 88% to 94%. LNPs were stored at −80 °C at an mRNA concentration of approximately 0.5 mg/mL.

### 2.3. Generation of Recombinant Proteins

The four candidate antigens have already been produced as recombinant proteins in previous works in our laboratory. Recombinant OeSOD, OmPLA2 and Om86 were expressed in the pQE30 vector and *Escherichia coli* M15 cells [21,22,24], whereas recombinant OeTSP1 was expressed in the pGEX-4T-1 vector and *E. coli* BL21 cells [23]. Recombinant firefly luciferase was purchased from Promega Biotech Ibérica (Madrid, Spain).

### 2.4. Immunisation of Rabbits

**Figure 1:**
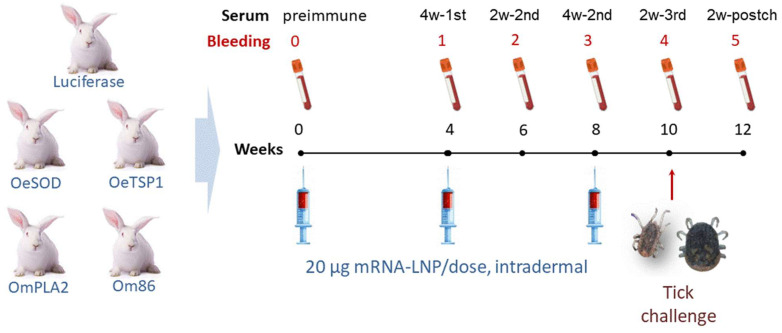
Immunisation of rabbits with *Ornithodoros* antigens or luciferase mRNA-LNPs. Groups of three animals each received three intradermal administrations of 20 µg mRNA-LNP per dose. Blood samples were collected prior to the first immunisation (week 0, pre-immune sera), four weeks after the first dose (week 4, 4w-1st sera), two and four weeks following the second dose (weeks 6 and 8, 2w-2nd and 4w-2nd sera), and two weeks after the third dose, which coincided with the tick challenge (week 10, 2w-3rd sera, red arrow). A final sample was obtained two weeks post-challenge (week 12, 2w-postch sera).

Five groups of three female New Zealand White rabbits each were immunised intradermally with 20 µg of the corresponding tick antigen mRNA-LNP or luciferase mRNA-LNP (control). The required amount of frozen mRNA-LNP was thawed at room temperature, diluted with sterile PBS up to a final volume of 200 µL. The preparation was then administered intradermally in four sites (50 µL per site) between the scapulae. Rabbits received two booster doses at four-week intervals, for a total of three mRNA-LNP administrations (Figure 1). This protocol of immunisation was based in previous works by Matias et al. [31,32,33,34].

Blood samples were collected at multiple time points: prior to the first immunisation (week 0, pre-immune sera), four weeks after the first dose (week 4, 4w-1st sera), two and four weeks after the second dose (weeks 6 and 8, 2w-2nd and 4w-2nd sera), two weeks after the third dose, coinciding with the tick challenge (week 10, 2w-3rd sera), and two weeks post-challenge (week 12, 2w-postch sera). Samples were allowed to clot at room temperature, and sera were separated and stored at −80 °C.

### 2.5. Humoral Response Analysis

To confirm that the rabbits had been immunised, the IgG antibody titre of sera collected two weeks after the third mRNA-LNP dose (week 10, 2w-3rd sera) was determined by serial dilution ELISA, following standard procedures described elsewhere [24]. Briefly, ELISA plates were coated with 100 ng/well of the homologous recombinant antigen. Sera were diluted in PBS supplemented with 0.05% Tween 20 (TPBS) in a twofold serial dilution from 1:100 to 1:12,800, and anti-rabbit IgG-horseradish peroxidase (Merck Life Science S.L.U., Madrid, Spain) was used at a 1:10,000 dilution. Orthophenylene-diamine was used as chromogenic substrate for peroxidase, the reaction was stopped with 3 N sulphuric acid, and optical density (OD) was measured at 492 nm. The antibody titre was defined as the highest serum dilution yielding a signal more than twice that of the corresponding pre-immune serum at the same dilution.

Subsequently, the evolution of IgM and IgG antibody levels -against the homologous recombinant antigens- throughout the experiment was assessed by ELISA following procedures similar to those described previously [24]. ELISA plates were coated with 100 ng/well of recombinant antigen, sera were tested at a 1:100 dilution, and anti-rabbit IgG and IgM antibodies were used at 1:10,000 and 1:5000 dilutions, respectively. Comparisons of OD values across different time points were performed using two-way ANOVA followed by Tukey’s multiple comparisons test, using GraphPad Prism 10 software (GraphPad Software 10.4.1).

Additionally, the IgG reactivity of immune sera collected two weeks after the third antigen dose (week 10, 2w-3rd sera) to saliva and midgut protein extracts from both *O. moubata* and *O. erraticus* was assessed by ELISA and Western blot, following standard procedures [24]. Sera were used at 1:100 and 1:50 dilutions for ELISA and Western blot, respectively, and anti-rabbit IgG was used at 1:10,000 for ELISA and at 1:2000 for Western blot. Reactive bands in Western blot were visualised using a chemiluminescent substrate (Clarity™ Western ECL Substrate, BIO-RAD laboratories, Alcobendas, Madrid, Spain).

### 2.6. Tick Challenge and Evaluation of Vaccine Efficacy

Two weeks after the final antigen dose (week 10), tick batches comprising 15 females, 30 males, and 50 third-stage nymphs (nymphs-3) of *O. erraticus* and *O. moubata* were allowed to feed on each rabbit for a maximum of two hours, although most ticks completed feeding in under one hour. The weight of unfed ticks was estimated by weighing each batch and dividing the total weight by the number of individuals in the batch.

Following feeding, ticks were maintained at 28 °C and 85% relative humidity for 24 h to allow for coxal fluid excretion. Subsequently, they were weighed in batches, and each female was placed in an individual plastic vial with two fed males to facilitate mating and reproduction. Nymphs-3 from the same rabbit were housed together in a single vial. All ticks were kept under controlled conditions (28 °C, 85% relative humidity, 12 h light–dark cycle).

As described in previous studies [24,25], vaccine effects on ticks were assessed by measuring the following parameters: (i) the quantity of blood ingested, calculated as the difference in weight before and 24 h after feeding; (ii) oviposition and fertility rates of females, expressed as the number of eggs laid per female and the subsequent number of newly hatched larvae per female (or nymphs-1 in the case of *O. moubata*); (iii) moulting rate of nymphs-3; and (iv) survival rates of all developmental stages two months after feeding on control and vaccinated rabbits.

Data for each parameter were summarised as mean ± standard deviation (SD) per group. Statistical differences between the control group and each vaccinated group were assessed by one-way ANOVA followed by Dunnett’s test. Differences were considered statistically significant at *p* < 0.05. All statistical analyses were conducted using SPSS v.29 software (IBM, Armonk, New York, USA).

Vaccine efficacy (E) for each antigen was calculated following the formula established by Contreras and de la Fuente [35], based on the reduction in developmental processes in ticks fed on vaccinated animals compared with those fed on controls. Specifically, efficacy was calculated as: E = 100 × [1 − (S × F × N × M)], where S and F represent the reductions in survival and fertility of females, and N and M represent reductions in survival and moulting of nymphs-3 [24,25].

Additionally, correlations between specific anti-antigen IgG antibody levels and tick parameters were assessed by simple linear regression analysis for each target antigen using GraphPad Prism 10 software.

## 3. Results

### 3.1. mRNA-LNPs Encoding Ornithodoros Antigens and Firefly Luciferase Induce Humoral Immune Responses in Rabbits

**Figure 2:**
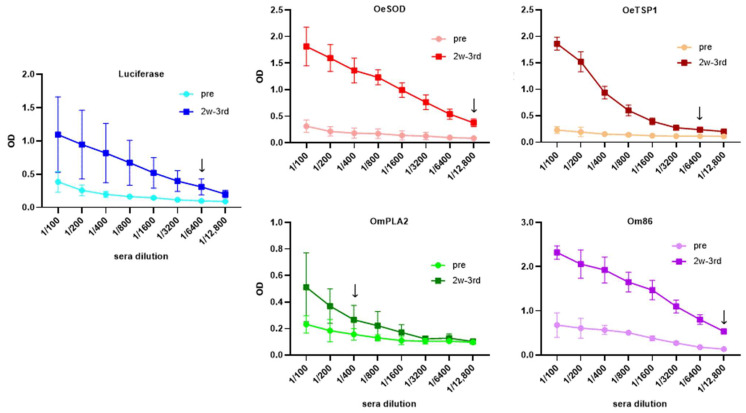
Humoral immune response elicited by mRNA-LNP vaccination. IgG antibody titres in rabbits immunised with three doses of mRNA-LNPs encoding luciferase (Luc), OeSOD, OeTSP1, OmPLA2, or Om86, measured by ELISA against the homologous recombinant antigens. Serum samples collected before immunisations (week 0, pre) and at two weeks after the third mRNA dose (week 10, 2w-3rd sera) were serially diluted from 1:100 to 1:12,800. Data represent the mean optical density (OD) values ± standard deviation (SD) at 492 nm for each rabbit group. The antibody titre was defined as the highest serum dilution yielding a signal more than twice that of the corresponding pre-immune serum at the same dilution. Black arrows indicate the average titre in each group.

Titration of IgG antibody levels in sera collected two weeks following administration of three mRNA-LNP doses (week 10, 2w-3rd sera) revealed that all five mRNA-LNP formulations -encoding tick antigens or firefly luciferase- elicited noticeable antibody responses in the immunised rabbits (Figure 2). IgG titres varied according to the mRNA-LNP construct, with average titres higher than 1:6400 for luciferase, 1:12,800 for both OeSOD and Om86, 1:6400 for OeTSP1, and higher than 1:400 for OmPLA2. Inter-individual variation was observed within the luciferase group, as rabbit number one exhibited a markedly lower titre of only 1:200.

**Figure 3:**
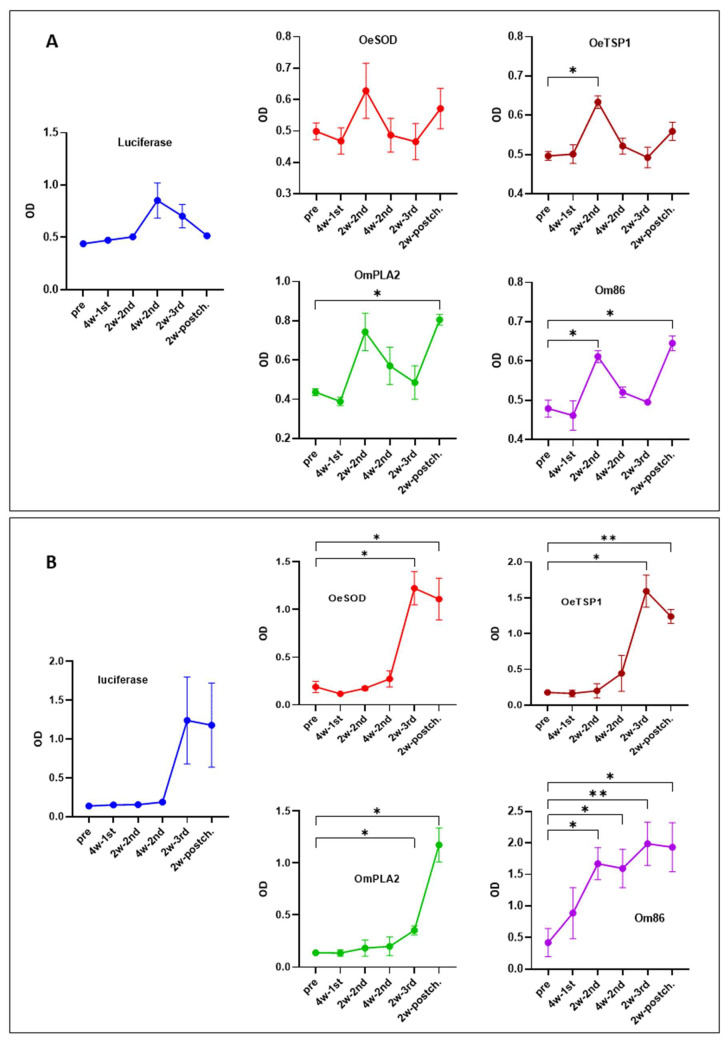
Kinetics of the humoral immune response elicited by mRNA-LNP vaccination. IgM (**A**) and IgG (**B**) antibody responses in rabbits immunised with mRNA-LNPs encoding luciferase, OeSOD, OeTSP1, OmPLA2, or Om86, measured by ELISA against the homologous recombinant antigens. Sera were collected at baseline (week 0, pre), four weeks after the first dose (week 4, 4w-1st sera), two and four weeks after the second dose (weeks 6 and 8, 2w-2nd and 4w-2nd sera), two weeks after the third dose (week 10, 2w-3rd sera), and two weeks post-challenge (week 12, 2w-postch sera). Serum samples were diluted 1:100; secondary antibodies were used at 1:5000 for IgM and 1:10,000 for IgG detection. Data represent the mean optical density (OD) values ± standard deviation (SD) at 492 nm for each rabbit group. Statistical differences between pre-immune sera and post-immunisation time points were assessed using two-way ANOVA followed by Tukey’s multiple comparisons test (* *p* < 0.05, ** *p* < 0.01).

Following titration, we assessed the levels of IgM and IgG antibodies to each antigen target throughout the experimental period (Figure 3).

The IgM response to the four tick antigens followed a similar kinetic pattern, reaching a first peak two weeks after the second mRNA-LNP dose (week 6), which was statistically significant for OeTSP1 and Om86, followed by a decline and a second peak two weeks after tick challenge (week 12) that was significant for OmPLA2 and Om86. In contrast, IgM antibodies against firefly luciferase peaked later, four weeks after the second mRNA-LNP dose (week 8), and then progressively declined until the end of the study (Figure 3A).

The IgG antibody kinetics, on the other hand, varied between mRNA groups (Figure 3B). IgG responses to luciferase, OeSOD, and OeTSP1 developed more slowly than IgM antibodies, remaining at low levels throughout the three-dose immunisation schedule and only peaking two weeks after the third dose (week 10), followed by a slight decrease post-tick challenge (week 12). Anti-OmPLA2 IgG antibodies required even more time to develop, reaching their maximum levels only after tick challenge (week 12). In contrast, anti-Om86 IgG antibodies appeared early, being detectable after a single mRNA-LNP dose, and increased progressively and significantly to reach a peak following the third dose.

These results are consistent with the antibody titres shown in Figure 2, confirming that all five mRNA-LNP constructs were successfully taken up and translated into proteins by rabbit cells. The resulting endogenously produced proteins were subsequently presented to the rabbit immune system, eliciting antigen-specific humoral responses of varying magnitude, ranging from low (OmPLA2) and moderate (OeTSP1, luciferase) to strong (OeSOD, Om86).

### 3.2. Humoral Responses Elicited by mRNA-LNPs Recognize Native Orthologous Proteins in Tick Saliva and Midgut Extracts

**Figure 4:**
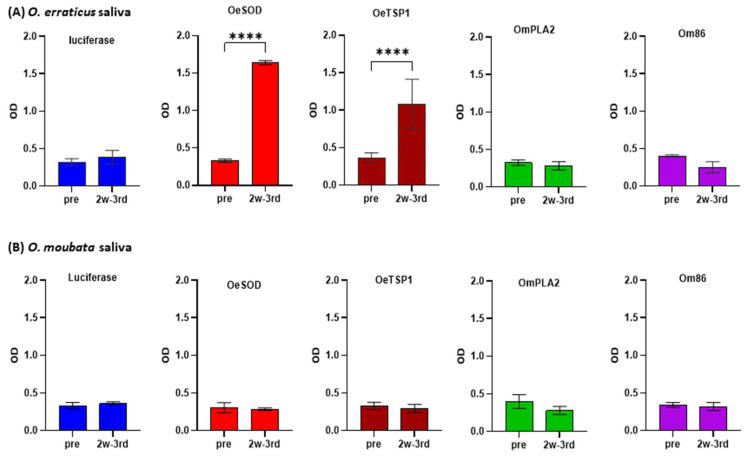
Reactivity of IgG antibody response against tick saliva. IgG antibody responses in rabbits immunised with mRNA-LNPs encoding luciferase, OeSOD, OeTSP1, OmPLA2, or Om86, measured by ELISA against the saliva of *O. erraticus* (**A**) and *O. moubata* (**B**). Sera collected before immunisation (week 0, pre) and two weeks after the third dose (week 10, 2w-3rd sera) were diluted 1:100 and anti-rabbit IgG was used at 1:10,000. Data represent the mean optical density (OD) values ± standard deviation (SD) at 492 nm for each rabbit group. Statistical differences between pre-immune and 2w-3rd sera were assessed using two-way ANOVA followed by Tukey’s multiple comparisons test (**** *p* < 0.0001).

**Figure 5:**
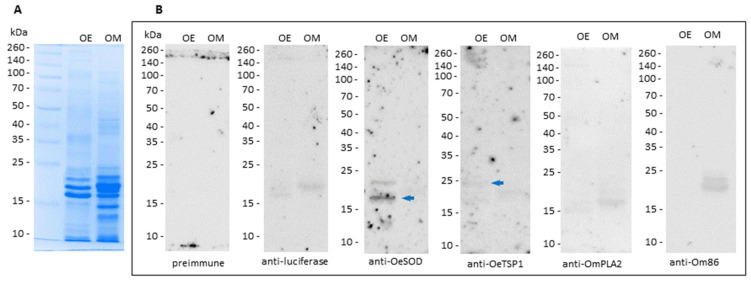
Proteins recognised on *Ornithodoros* tick saliva by the IgG antibody responses. (**A**) Coomassie Blue-stained 12% SDS-PAGE gel showing saliva from *O. erraticus* (OE) and *O. moubata* (OM) female ticks. (**B**) Western blots showing antigens detected by sera from rabbits immunised with mRNA-LNPs collected two weeks after the third mRNA-LNP dose (week 10, 2w-3rd sera). Arrow indicates native forms of OeSOD. kDa, molecular weight in kilodaltons.

Once confirmed that the mRNA-based vaccines induced humoral immune responses, the reactivity of the IgG antibody responses elicited by three doses of mRNA-LNPs (namely, reactivity of the 2w-3rd sera, collected at week 10) against the saliva of both *Ornithodoros* species was assessed by ELISA (Figure 4) and Western blot (Figure 5).

As expected, IgG antibodies induced by luciferase mRNA did not recognise any antigens in the saliva samples. By contrast, IgG antibodies generated in response to mRNAs encoding *O. erraticus* antigens OeSOD and OeTSGP1 reacted significantly with *O. erraticus* saliva, but showed no reactivity towards *O. moubata* saliva (Figure 4). These results were also observed on the Western blot, where bands compatible in size with OeSOD (clear) and OeTSP1 (faint) were revealed on the *O. erraticus* saliva, but no perceptible bands were observed on *O. moubata* saliva (Figure 5). These findings confirm the presence of native OeSOD and OeTSP1 in *O. erraticus* saliva. Given that OeTSP1 is a membrane protein, its presence in the salivary fluid was somewhat unexpected, but it cannot be discarded because tetraspanins can be secreted to tick saliva as part of the membrane of salivary exosomes [36,37].

IgG antibodies raised against *O. moubata* antigens OmPLA2 and Om86 did not show reactivity in ELISA or Western blot towards *O. erraticus* saliva. Anti-Om86 antibodies also failed to react with *O. moubata* saliva, which is consistent with the exclusive midgut expression of the Om86 protein. Unexpectedly, anti-OmPLA2 IgG antibodies did not detect the native protein in *O. moubata* saliva in ELISA or Western blot (Figure 4 and Figure 5). We hypothesise that this absence of signal may be attributable to the low anti-OmPLA2 antibody titre (1/400) in sera collected two weeks after the third mRNA-LNPs dose (Figure 2 and Figure 3).

**Figure 6:**
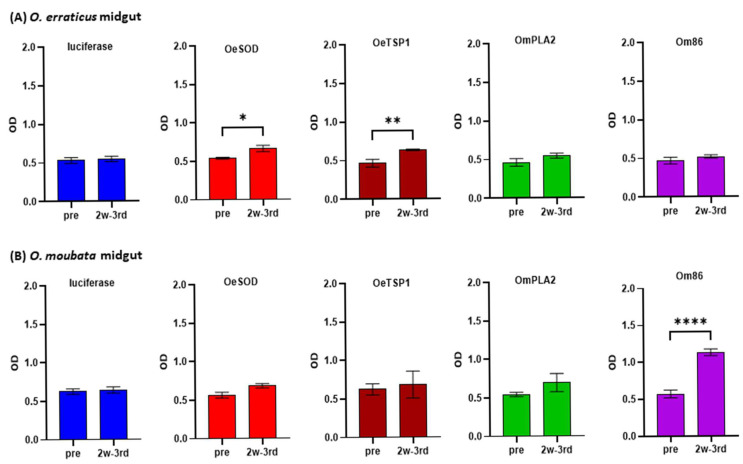
Reactivity of IgG antibody response against tick midgut. IgG antibody responses in rabbits immunised with mRNA-LNPs encoding luciferase, OeSOD, OeTSP1, OmPLA2, or Om86, measured by ELISA against the midgut protein extracts of *O. erraticus* (**A**) and *O. moubata* (**B**). Sera collected before immunisation (week 0, pre) and two weeks after the third dose (week 10, 2w-3rd sera) were diluted 1:100 and anti-rabbit IgG was used at 1:10,000. Data represent the mean optical density (OD) values ± standard deviation (SD) at 492 nm for each rabbit group. Statistical differences between pre-immune and 2w-3rd sera were assessed using two-way ANOVA followed by Tukey’s multiple comparisons test (* *p* > 0.05, ** *p* < 0.001, **** *p* < 0.0001).

**Figure 7:**
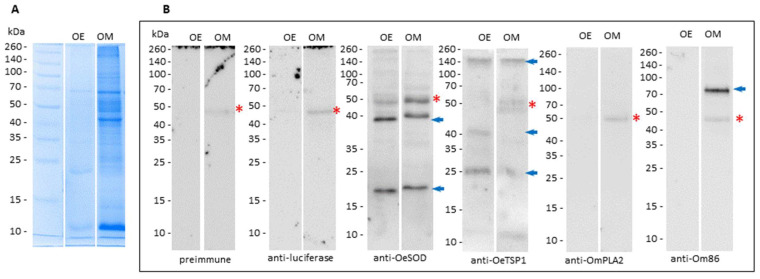
Proteins recognised on *Ornithodoros* tick midgut extracts by the IgG antibody responses. (**A**) Coomassie Blue-stained 12% SDS-PAGE gel showing midgut extracts from *O. erraticus* (OE) and *O. moubata* (OM) female ticks. (**B**) Western blots showing antigens detected by sera from rabbits immunised with mRNA-LNPs collected two weeks after the third mRNA-LNP dose (week 10, 2w-3rd sera). Red asterisks: remains of IgG heavy chain from the rabbit host ingested with blood. Arrows indicate native forms of OeSOD, OeTSP1 and Om86. kDa, molecular weight in kilodaltons.

Likewise, the reactivity of the 2w-3rd sera (collected at week 10) against midgut extracts of both *Ornithodoros* species was also assessed by ELISA (Figure 6) and Western blot (Figure 7). High background reactivity in both ELISA and Western blot assays was attributed to non-specific recognition of dietary rabbit IgG within these extracts, as revealed by pre-immune and anti-luciferase sera.

Anti-OeSOD and anti-OeTSGP1 sera exhibited slight reactivity in ELISA to midgut extracts from both *Ornithodoros* species, which reached statistical significance only for *O. erraticus* midgut (Figure 6). In Western blot, anti-OeSOD sera recognised two bands of approximately 19 and 40 kDa in *O. erraticus* likely corresponding to monomeric and dimeric forms of native SOD [24], and two similar, though markedly fainter, bands in *O. moubata* (Figure 7). Anti-OeTSP1 sera detected in *O. erraticus* three bands consistent with the monomeric (25 kDa) and oligomeric (40 and 140 kDa) forms of tetraspanins, in agreement with previous findings by our group [23], as well as two weaker bands of 25 kDa and 140 kDa in *O. moubata*. These results confirm the expression of OeSOD and OeTSGP1 in the midgut of *O. erraticus* and suggest the existence of shared epitopes with orthologous midgut proteins in *O. moubata*.

Anti-OmPLA2 sera displayed low reactivity in ELISA against both *O. moubata* and *O. erraticus* midgut extracts (Figure 6), and did not reveal any detectable bands in the Western blot (Figure 7). In contrast, anti-Om86 IgG sera showed significant reactivity in ELISA to *O. moubata* midgut extracts (Figure 6) and detected a well-defined band close to 85 kDa, consistent with the expected molecular weight of Om86 (Figure 7). Conversely, anti-Om86 sera showed negligible reactivity in ELISA against the midgut extract of *O. erraticus* and failed to detect any clear band in this species, consistent with previous findings reported by our group [22].

Taken together, these results indicate that immunisation of rabbits with the tick mRNA-LNP constructs induces antigen-specific IgG antibody responses, which -except anti-OmPLA2- are capable of recognising their respective target proteins in their native form within tick salivary and midgut tissues.

Luciferase mRNA-LNP was used as a non-related antigen control to verify the integrity of the formulation and the efficiency of in vivo expression. The high anti-luciferase antibody levels reached in two of the three rabbits immunised with luciferase mRNA-LNP confirmed that this platform effectively delivered and expressed the control protein. As expected, this control did not elicit IgG responses against tick antigens nor did it influence tick biological parameters (see below).

### 3.3. Protective Effects of the Immune Response on the Ticks

**Figure 8:**
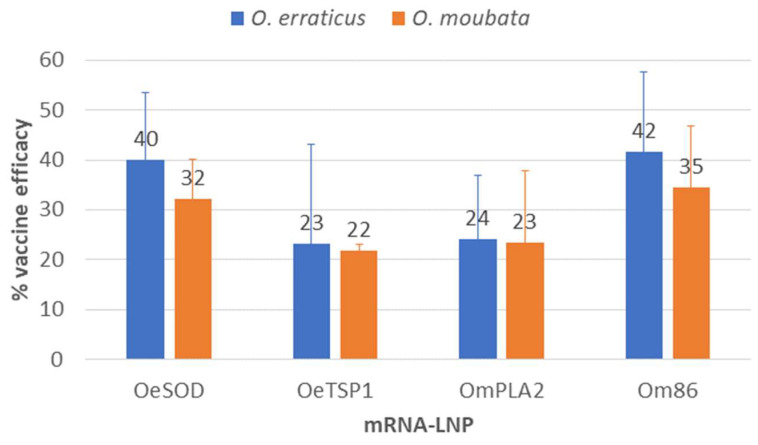
Vaccine efficacy. Vaccine efficacy of the mRNA-LNP constructs encoding tick antigens (OeSOD, OeTSP1, OmPLA2 and Om86) calculated as E = 100 × [1 − (S × F × N × M)], where S and F represent the reductions in survival and fertility of females, and N and M represent reductions in survival and moulting of nymphs-3.

In *O. erraticus* ticks (Table 2), the immune response elicited by mRNA-LNPs encoding tick antigens resulted in: (i) a general trend towards reduced blood intake across all developmental stages, reaching statistical significance in males fed on rabbits vaccinated with OmPLA2, females fed on rabbits vaccinated with Om86, and nymphs-3 fed on rabbits vaccinated with OeTSP1; (ii) overall, though modest and statistically non-significant, reductions in nymphs-3 moulting rates; and (iii) statistically significant reductions in female oviposition and fertility, ranging from 17.6% to 39.7%. Overall, the significant reductions observed in tick biological parameters correlated significantly with specific IgG antibody levels, particularly for reproductive traits such as female oviposition and fertility. For these parameters, Pearson’s correlation coefficient (r) ranged from −0.83 to −0.97, indicating a very strong correlation between antibody levels and reductions in tick parameters (Supplementary Figure S1). These effects translated into vaccine efficacies against *O. erraticus* of 40.0% (OeSOD), 23.1% (OeTSP1), 24.1% (OmPLA2), and 41.6% (Om86) (Figure 8).

In *O. moubata* ticks (Table 3), the effects of vaccination with mRNA-LNPs were broadly similar to those observed in *O. erraticus*, including: (i) non-significant reductions in feeding by males (in the OeTSP1, OmPLA2, and Om86 groups) and females (in the OeSOD, OeTSP1, and Om86 groups); (ii) general reductions in nymphs-3 moulting, statistically significant only in the OmPLA2 group; (iii) significant reductions in female oviposition and fertility in all groups except OeTSP1; and, in contrast to *O. erraticus*, (iv) generalised but modest reductions in survival across all developmental stages and antigen groups, which reached statistical significance only in females fed on rabbits vaccinated with OeTSP1 and Om86. As for *O. erraticus*, in general, reductions in the *O. moubata* biological parameters analysed showed a strong or very strong correlation with the specific IgG antibody levels, with Pearson’s correlation coefficient ranging from −0.6 to −0.97 (Supplementary Figure S2). Consequently, vaccine efficacies against *O. moubata* reached 32.1% (OeSOD), 21.9% (OeTSP1), 23.5% (OmPLA2), and 34.5% (Om86) (Figure 8).

Table 4 summarises the comparative vaccine efficacies achieved with the recombinant proteins and the mRNA-LNP formulations of the four antigenic targets. The mRNA-LNP constructs of OeSOD and OeTSP1 conferred, respectively, 26% and 50% lower protection than their recombinant counterparts against *O. erraticus*, although protection against *O. moubata* increased markedly, by 10-fold (OeSOD) and 3-fold (OeTSP1). The OmPLA2 mRNA-LNP conferred approximately 24% protection against both *Ornithodoros* species. This represented a 47% reduction in efficacy against *O. moubata*, but also demonstrated that OmPLA2 can induce significant cross-protection against *O. erraticus*, a property that had not been assessed for the recombinant OmPLA2 [21]. Finally, the Om86 mRNA-LNP elicited 41-fold greater cross-protection against *O. erraticus* and 5-fold greater protection against *O. moubata* than the recombinant Om86.

## 4. Discussion

This study demonstrates, for the first time, the immunogenicity and protective efficacy of mRNA-LNP vaccines targeting argasid ticks, specifically *O. erraticus* and *O. moubata*. Building on previous work with recombinant proteins [21,22,23,24], we reformulated four partially protective antigens -two salivary (OeSOD, OmPLA2) and two midgut-associated (OeTSP1, Om86)- as codon-optimised mRNA molecules encapsulated in lipid nanoparticles. These mRNA-LNP constructs elicited robust antigen-specific IgG responses and conferred moderate levels of protection against both *O. erraticus* and *O. moubata*, with efficacy values ranging from 21.9% to 41.6%.

Consistent with previous reports on mRNA-based vaccines against *I. scapularis* [31,32,33,34], our results confirm that the mRNA-LNP platform is capable of inducing specific humoral responses in lagomorphs. Antigen-specific IgG responses peaked after the third dose and were particularly pronounced for OeSOD and Om86, mirroring the pattern observed for luciferase, the unrelated control antigen. These responses were corroborated by reactivity against native tick salivary and midgut extracts, as demonstrated by ELISA and Western blot. Therefore, the antibodies induced by mRNA-LNPs not only recognised the homologous recombinant antigens used in ELISA but also detected their native counterparts in *Ornithodoros* tissues, indicating appropriate protein translation and antigen processing in vivo. These findings are significant, as the preservation of conformational and glycosylation-dependent epitopes is a known limitation of recombinant antigens produced in prokaryotic systems [27,28,29,30], and one that mRNA-LNPs may overcome.

As in previous studies with recombinant proteins [26,27], vaccine-induced antibody levels showed strong and significant correlations (*r* < −0.6, *p* < 0.05) with reductions in tick feeding, reproduction, and development, particularly with female oviposition and fertility, two parameters directly associated with population dynamics (supplementary Figures S1 and S2). Although the overall reductions in tick bloodmeal size, moulting, and survival were modest, the consistent and statistically significant impact on reproductive parameters highlights the value of targeting antigens involved in female physiology and midgut function.

Interestingly, differences in antigen performance were observed between species and platforms. For example, while recombinant OeSOD and OeTSP1 induced higher protection against *O. erraticus* than their mRNA-LNP counterparts (54–56% vs. 23–40%) (Table 4), the opposite trend was observed for *O. moubata*, where mRNA-LNPs yielded substantially improved protection (32–34%) over the negligible efficacy of recombinant OeSOD (3.1%) and OeTSP1 (11.1%). This finding suggests that mRNA-LNP vaccination may not only preserve structural epitopes better than recombinant proteins, but may also broaden cross-species protective immunity, potentially through enhanced antigen folding or glycosylation patterns that improve immune recognition across orthologues.

In contrast, the OmPLA2 mRNA-LNP induced relatively modest IgG titres and provided limited protection (~24%) against both *Ornithodoros* species. While this represents a reduction in efficacy against *O. moubata* compared to the recombinant OmPLA2 (44.2%), it suggests cross-protection against *O. erraticus* for this antigen (24.1%). These results show that vaccination with mRNA-LNP encoding OmPLA2 elicited antibodies that were associated with reduced tick parameters in both *O. erraticus* and *O. moubata*. This finding is consistent with an antigen-driven effect (i.e., conservation of epitopes between orthologous OmPLA2), but does not by itself demonstrate a platform-specific advantage in the absence of a head-to-head comparison with the recombinant OmPLA2 antigen for *O. erraticus*. The relatively low titres observed for OmPLA2 may reflect suboptimal antigen expression or immunogenicity, warranting further investigation into mRNA design, immunization protocols, and direct comparative studies with the recombinant protein.

The most striking improvement was observed with the Om86 mRNA-LNP, which conferred a 5-fold increase in efficacy against *O. moubata* and an unprecedented 41-fold increase in cross-protection against *O. erraticus* compared to its recombinant form. As Om86 is a homologue of the well-characterised Bm86 antigen used in commercial anti-*Rhipicephalus* vaccines, its success in this study reinforces the potential for conserved midgut antigens to serve as broad-spectrum vaccine targets [38,39,40]. It also supports the hypothesis that mRNA-LNPs may enhance antigen folding and presentation in a way that unmasked cryptic or conformational epitopes not accessible in *E. coli*-derived recombinant proteins.

Beyond efficacy, the practical advantages of mRNA-LNP vaccines are particularly relevant for tick control. These include rapid production, flexible antigen design, and the absence of material derived from prokaryotic expression cells, which collectively enable a faster and safer response to emerging tick-borne threats. Given the environmental and resistance-related limitations of chemical acaricides [10,11], mRNA-LNP vaccines could become a cornerstone of integrated tick management, particularly in settings where *Ornithodoros* spp. maintain zoonotic pathogens such as African swine fever virus and *Borrelia* spp. [3,5,6].

Nevertheless, several challenges must be addressed before field application. These include the need to improve consistency of immune responses across individuals, optimise dose and delivery routes for field-relevant hosts, and extend protective efficacy beyond laboratory models. The kinetics of IgM and IgG responses observed in this study also suggest the potential benefit of refining immunisation schedules to maximise long-term immunity and memory responses.

In conclusion, this study provides compelling evidence for the utility of mRNA-LNP vaccines targeting *Ornithodoros* tick antigens. The platform offers promising improvements in antigen-specific immunity, functional protection, and cross-species efficacy compared to traditional protein-based vaccines. These results pave the way for further exploration of mRNA technologies in the context of argasid tick control and the prevention of tick-borne diseases of medical and veterinary importance.

## Figures and Tables

**Table 1:** Salivary and intestinal antigens from *Ornithodoros erraticus* and *Ornithodoros moubata* selected for formulation as mRNA-LNPs. Vaccine efficacy (% E) provided by each antigen in recombinant form against *O. erraticus* and *O. moubata* in previous studies. SG, salivary glands; MG, midgut. ND, not determined.

Antigen Name	Protein Description	GenBank/ Uniprot Code	Species	Organ	% E *O. erraticus*	%E *O. moubata*	References
OeSOD	Superoxide dismutase	MBZ4008920.1	*O. erraticus*	SG	54.3	3.1	[24]
OeTSP1	Tetraspanin	A0A293MYE4	*O. erraticus*	MG	56.0	11.1	[23]
OmPLA2	Phospholipase A2	AGJ90343.1	*O. moubata*	SG	ND	44.2	[21]
Om86	Bm86 antigen homologue	A0AA96JYS1	*O. moubata*	MG	0	7	[22]
Luc	Luciferase	BAF48396.1	*P. pyralis*	-	-	-	[34]

**Table 2:** Effect of the vaccination with OeSOD, OsTSP1, OmPLA2 and Om86 mRNA-LNPs on *Ornithodoros erraticus* ticks fed on control and vaccinated rabbits. Control rabbits were administered *Photinus pyralis* luciferase (LUC) mRNA-LNP. Results are shown as mean ± standard deviation for each rabbit group. Means were compared between ticks fed on vaccinated and control rabbits by one-way ANOVA followed by the Dunnett’s *t*-test. In parentheses, % of reduction in the corresponding parameter respect to the control. Values of *p* < 0.05 were considered significant (* *p* < 0.05, ** *p* < 0.01).

Parameter	Developmental Stage	LUC (Control)	OeSOD (% Reduction)	OeTSP1 (% Reduction)	OmPLA2 (% Reduction)	Om86 (% Reduction)
Ingested blood (mg)	Males	3.8 ± 0.2	3.0 ± 1.0 (20.1)	3.3 ± 0.2 (11.8)	3.0 ± 0.1 (20.7) *	3.5 ± 0.7 (7.8)
	Females	13.0 ± 0.8	13.6 ± 1.9	11.1 ± 1.1 (14.1)	13.8 ± 1.2	11.5 ± 0.3 (11.7) *
	Nymphs-3	2.6 ± 0.1	2.4 ± 0.3 (5.9)	2.2 ± 0.1 (14.6) *	2.4 ± 0.3 (5.9)	2.6 ± 0.3
Survival (%)	Males	100 ± 0	100 ± 0	100 ± 0	100 ± 0	99.9 ± 1 (0.1)
	Females	100 ± 0	100 ± 0	100 ± 0	100 ± 0	100 ± 0
	Nymph-3	99.0 ± 1.0	98.6 ± 1.0 (0.4)	100 ± 0	99.3 ± 1.0	99.3 ± 1.0
Moulting (%)	Nymphs-3	98.6 ± 2.4	90.2 ± 6.4 (8.4)	89.8 ± 14.4 (8.8)	92.6 ± 6.5 (6.0)	90.3 ± 5.3 (8.3)
Oviposition (eggs/female)	Females	52.1 ± 2.4	36.9 ± 4.1 (29.2) *	39.6 ± 2.7 (24.0) *	42.5 ± 6.1 (18.4) *	31.4 ± 9.1 (39.7) **
Fertility (larvae/female)	Females	44.2 ± 1.4	28.7 ± 4.2 (35.1) **	36.4 ± 2.8 (17.6) *	35.2 ± 3.4 (20.4) *	28.4 ± 8.5 (35.7) **
Efficacy (%)			40 ± 11.9	23.1 ± 15.5	24.1 ± 10.9	41.6 ± 13.6

**Table 3:** Effect of the vaccination with OeSOD, OsTSP1, OmPLA2 and Om86 mRNA-LNPs on *Ornithodoros moubata* ticks fed on control and vaccinated rabbits. Control rabbits were administered *Photinus pyralis* luciferase (LUC) mRNA-LNP. Results are shown as mean ± standard deviation for each rabbit group. Means were compared between ticks fed on vaccinated and control rabbits by one-way ANOVA followed by the Dunnett’s *t*-test. In parentheses, % of reduction in the corresponding parameter respect to the control. Values of *p* < 0.05 were considered significant (* *p* < 0.05, ** *p* < 0.01).

Parameter	Developmental Stage	LUC (Control)	OeSOD (% Reduction)	OeTSP1 (% Reduction)	OmPLA2 (% Reduction)	Om86 (% Reduction)
Ingested blood (mg)	Males	32.1 ± 1.8	33.1 ± 3.4	28.0 ± 2.0 (11.8)	29.2 ± 3.0 (9.0)	29.2 ± 1.2 (8.9)
	Females	216.2 ± 1.1	187.6 ± 40.8 (13.2)	201.6 ± 1.0 (6.8)	230.5 ± 20.4	213.4 ± 18.7 (1.3)
	Nymphs-3	12.3 ± 3.1	12.3 ± 2.4	13.5 ± 0.9	15.5 ± 1.4	12.3 ± 1.6
Survival (%)	Males	96.4 ± 3.6	92.2 ± 8.7 (4.2)	92.6 ± 3.3 (3.8)	96.5 ± 0.2	96.3 ± 0.1 (0.1)
	Females	96.7 ± 03.3	88.9 ± 8.3 (4.5)	80 ± 1.0 (16.7) *	91.1 ± 8.3 (5.6)	77.8 ± 8.3 (18.9) **
	Nymph-3	98.9 ± 1.1	93.3 ± 5.0 (5.6)	97.0 ± 1.0 (1.9)	94.0 ± 7.1 (4.9)	90.5 ± 6.3 (8.4)
Moulting (%)	Nymphs-3	97.9 ± 0.1	96.5 ± 62.2 (1.4)	96.9 ± 1.5 (1.0)	93.5 ± 3.8 (4.4) *	97.1 ± 1.1 (0.8)
Oviposition (eggs/female)	Females	258.0 ± 0.7	196.8 ± 34.9 (23.7) **	228.6 ± 13.8 (11.4)	219.0 ± 9.6 (15.1) **	221.7 ± 17.2 (14.1) **
Fertility (larvae/female)	Females	228.8 ± 0.41	184.0 ± 28.7 (19.6) **	222.4 ± 2.3 (2.8)	204.1 ± 12.2 (10.8) *	203.3 ± 9.9 (11.1) *
Efficacy (%)			32.1 ± 7.2	21.9 ± 1.0	23.5 ± 12.3	34.6 ± 10.4

**Table 4:** Vaccine efficacy of the four *Ornithodoros* target antigens administered as recombinant proteins or mRNA-LNPs. Red and blue arrows indicate lower or higher vaccine efficacy of mRNA-LNP construction respect to the recombinant antigen.

Antigen Name	Protein Description	Protective Efficacy Against *O. erraticus*		Protective Efficacy Against *O. moubata*	
		Recombinant	mRNA-LNP	Recombinant	mRNA-LNP
OeSOD	Superoxide dismutase	54.3%	40% 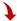	3.1%	32.1% 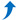
OeTSP1	Tetraspanin	56.0%	23.1% 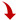	11.1%	21.9% 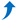
OmPLA2	Phospholipase A2	not determined	24.1%	44.2%	23.5% 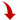
Om86	Bm86 antigen homologue	0%	41.6% 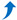	7%	34.6% 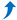

## Data Availability

The *Ornithodoros erraticus* and *Ornithodoros moubata* salivary and midgut transcriptomic data are available under Bioproject numbers PRJNA401392, PRJNA666995, RJNA667315 and PRJNA377416, respectively.

## Supplementary Materials

The following supporting information can be downloaded at: https://www.mdpi.com/article/10.3390/pathogens14090914/s1, Supplementary Figure S1: Simple linear regressions between IgG antibody levels against OeSOD, OeTSP1, OmPLA2, or Om86, induced by three doses of mRNA-LNPs (2-week post-third dose sera), and the parameters measured in *Ornithodoros erraticus* (Oe) ticks fed on control rabbits (vaccinated with firefly luciferase mRNA-LNP) and on rabbits vaccinated with each tick mRNA-LNP. The *X*-axis shows the optical density (OD) values at 492 nm for each rabbit serum sample diluted 1:100. The *Y*-axis shows the corresponding tick parameter values. Linear regression analyses were performed using GraphPad Prism version 10. Statistically significant regressions are highlighted in blue (* *p* < 0.05, ** *p* < 0.01). Supplementary Figure S2. Simple linear regressions between IgG antibody levels against OeSOD, OeTSP1, OmPLA2, or Om86, induced by three doses of mRNA-LNPs (2-week post-third dose sera), and the parameters measured in *Ornithodoros moubata* (Om) ticks fed on control rabbits (vaccinated with firefly luciferase mRNA-LNP) and on rabbits vaccinated with each tick mRNA-LNP. The *X*-axis shows the optical density (OD) values at 492 nm for each rabbit serum sample diluted 1:100. The *Y*-axis shows the corresponding tick parameter values. Linear regression analyses were performed using GraphPad Prism version 10. Statistically significant regressions are highlighted in blue (* *p* < 0.05, ** *p* < 0.01).
