# Supplementary material for: Immunization with mRNA-LNPs Encoding Ornithodoros Argasid Tick Antigens Induces Humoral Immune Responses and Tick Resistance
Source: Pathogens. 2025 Sep 11;14(9):914. doi: 10.3390/pathogens14090914 (PMC12472351; doi:10.3390/pathogens14090914)

Supplementary Figure S2. Simple linear regressions between IgG antibody levels against OeSOD, OeTSP1, OmPLA2, or Om86, induced by three doses of mRNA-LNPs (2-week post-third dose sera), and the parameters measured in *Ornithodoros moubata* (Om) ticks fed on control rabbits (vaccinated with firefly luciferase mRNA-LNP) and on rabbits vaccinated with each tick mRNA-LNP. The X-axis shows the optical density (OD) values at 492 nm for each rabbit serum sample diluted 1:100. The Y-axis shows the corresponding tick parameter values. Linear regression analyses were performed using GraphPad Prism version 10. Based on the results of the linear regression analysis, the magnitude of the Pearson correlation coefficient (r) was calculated: the sign of r corresponds to the slope, and the magnitude of r is the square root of R^{2}. Statistically significant regressions are highlighted in blue (*P < 0.05, **P < 0.01).

A. Linear regressions between anti-OeSOD IgG levels and *O. moubata* tick parameters.

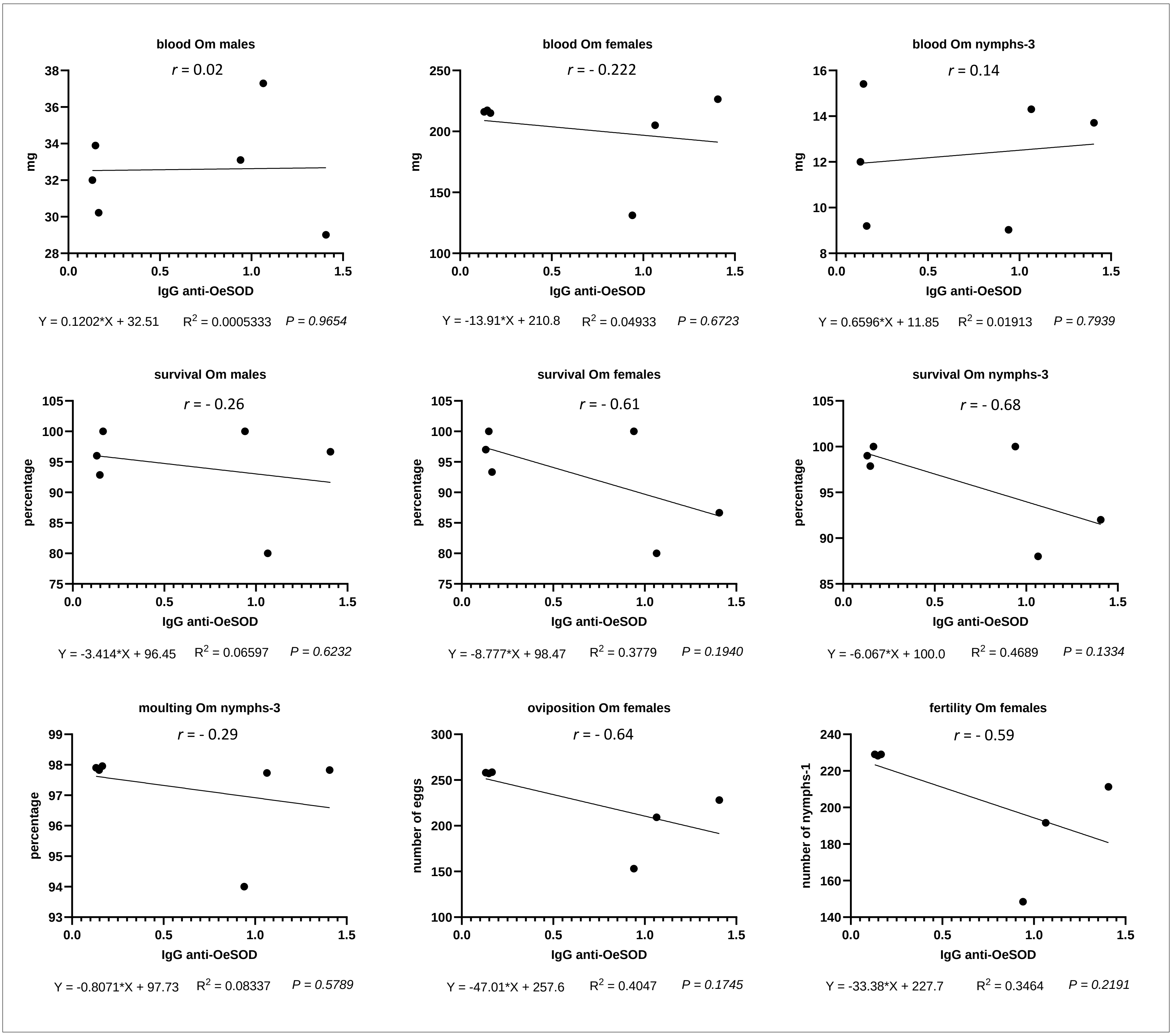

B. Linear regressions between anti-OeTSP1 IgG levels and *O. moubata* tick parameters.

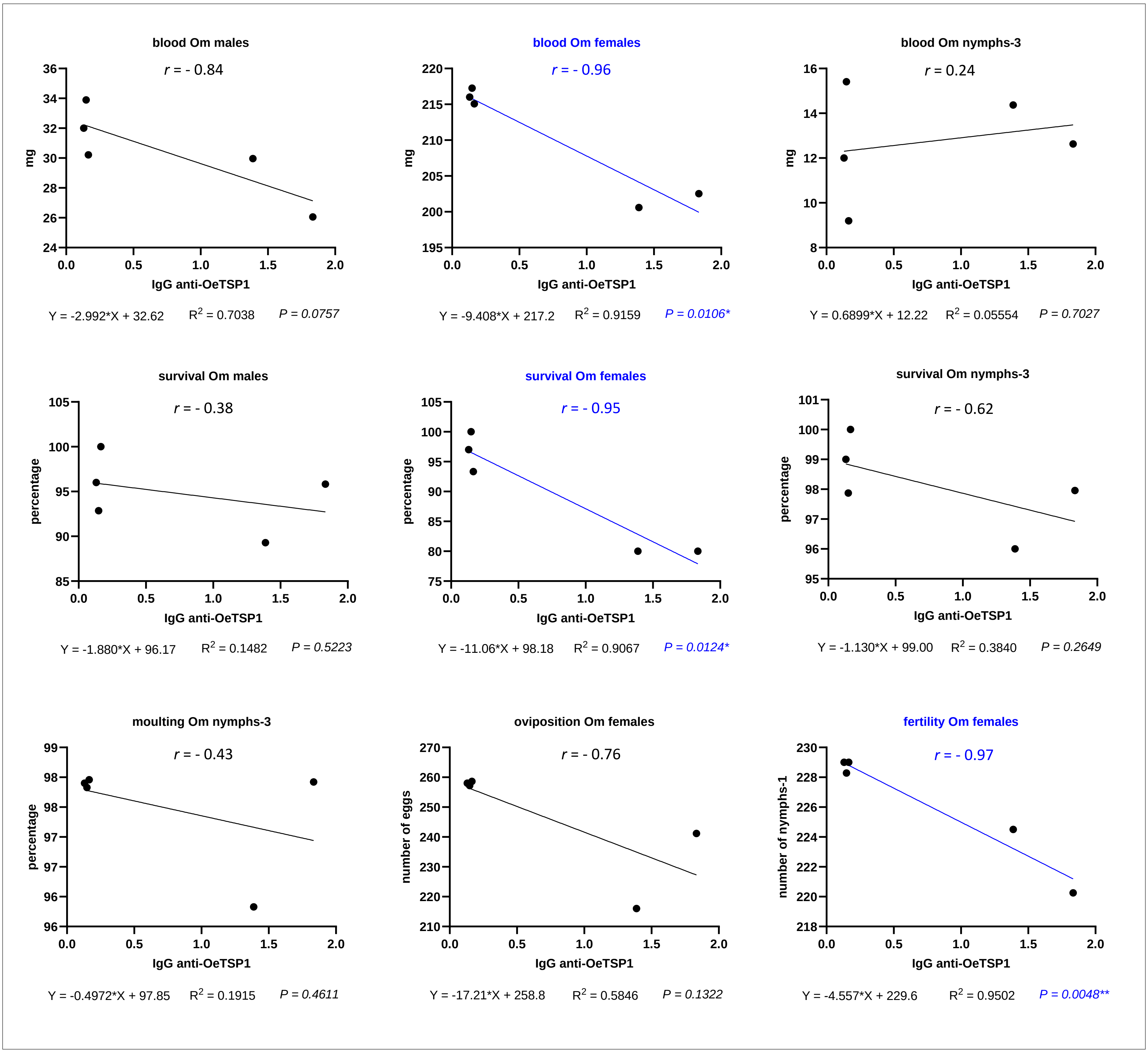

C. Linear regressions between anti-OmPLA2 IgG levels and *O. moubata* tick parameters.

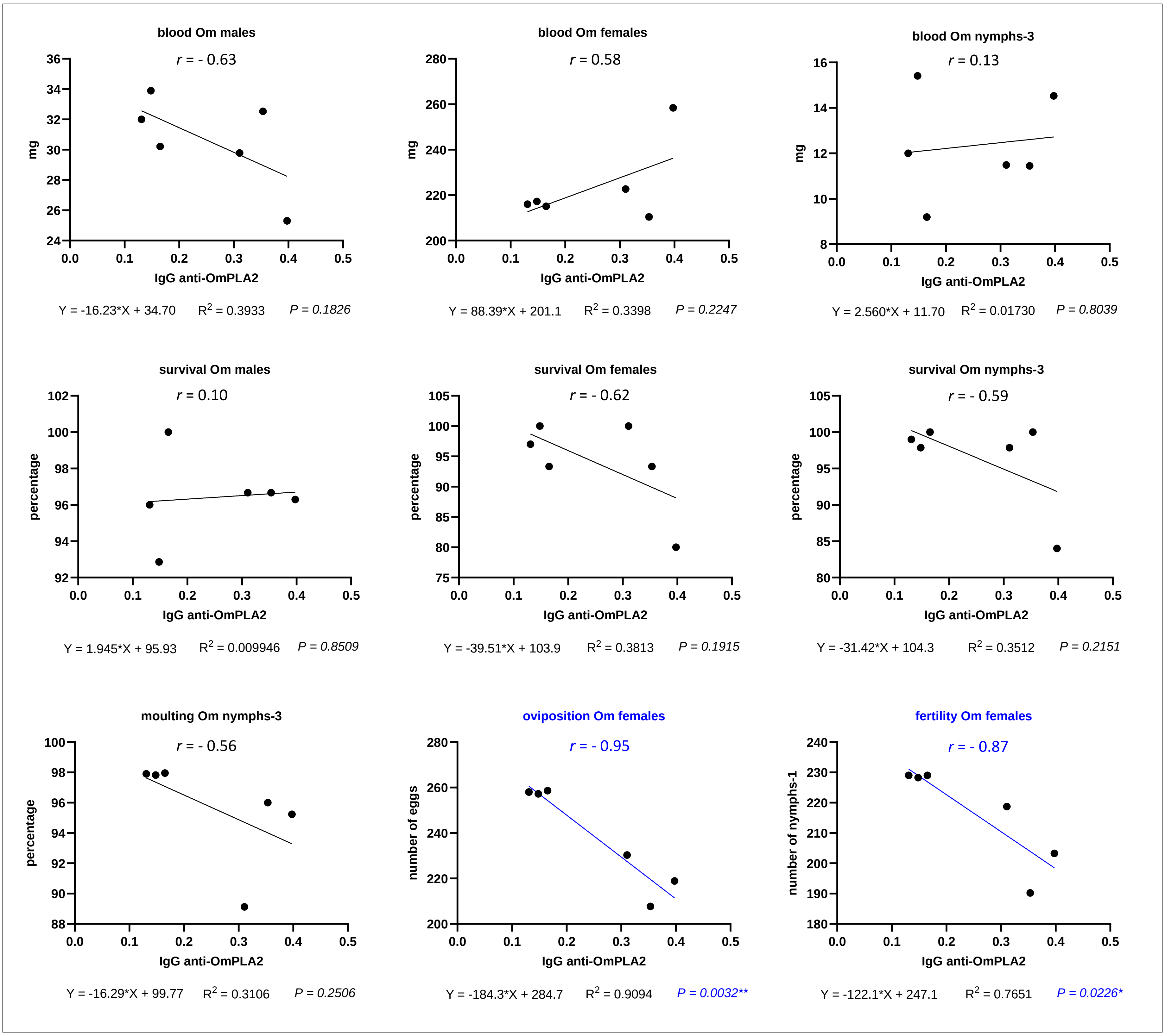

D. Linear regressions between anti-Om86 IgG levels and *O. moubata* tick parameters.

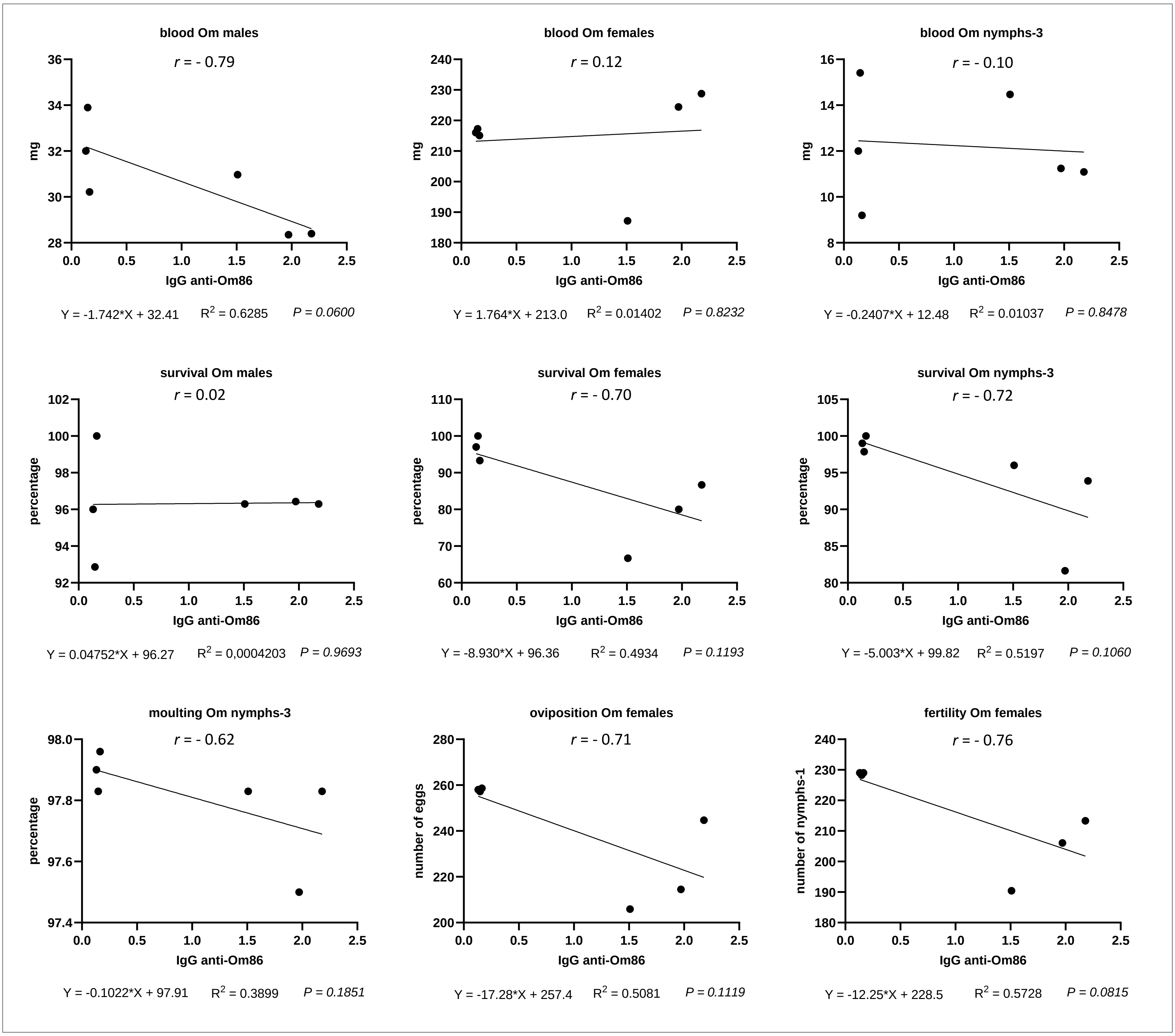

Supplement: Supplementary file 1 [file pathogens-14-00914-s001.zip › Supplementary Figure S2.pdf]
